# Reduced Local Symmetry in Lithium Compound Li_2_SrSiO_4_ Distinguished by an Eu^3+^ Spectroscopy Probe

**DOI:** 10.1002/advs.201802126

**Published:** 2019-05-20

**Authors:** Lei Chen, Peng Cheng, Zhao Zhang, Liangrui He, Yang Jiang, Guobao Li, Xiping Jing, Yan'guang Qin, Min Yin, Ting‐Shan Chan, Bin Hong, Shi Tao, Wangsheng Chu, Zhi Zhao, Haiyong Ni, Holger Kohlmann, Oliver Oeckler

**Affiliations:** ^1^ School of Materials Science and Engineering Hefei University of Technology Hefei 230009 China; ^2^ Intelligent Manufacturing Institute of Hefei University of Technology Hefei 230051 China; ^3^ College of Chemistry and Molecular Engineering Peking University Beijing 100871 China; ^4^ Department of Physics University of Science and Technology of China Hefei 230026 China; ^5^ National Synchrotron Radiation Research Center Hsinchu 30076 Taiwan; ^6^ Hefei Innovation Research Institute Beihang University Heifei 230013 China; ^7^ National Synchrotron Radiation Laboratory University of Science and Technology of China Hefei 230026 China; ^8^ Hefei National Laboratory for Physical Sciences at the Microscale University of Science and Technology of China Hefei 230026 China; ^9^ Guangdong Research Institute of Rare Metals Guangdong Academy of Sciences Guangzhou 510651 China; ^10^ Institut für Anorganische Chemie Universität Leipzig Johannisallee 29 04103 Leipzig Germany; ^11^ Institut für Mineralogie Kristallographie und Materialwissenschaft Universität Leipzig Scharnhorststr. 20 D‐04275 Leipzig Germany

**Keywords:** crystal structures, Eu^3+^ spectroscopy probes, lithium compounds, luminescence, symmetry breaking

## Abstract

Research on lithium compounds has attracted much attention nowadays. However, to elucidate the precise structure of lithium compounds is a challenge, especially when considering the small ions that may be transferred between the interstitial voids. Here, the discovery of reduced local symmetry (symmetry breaking) in small domains of Li_2_SrSiO_4_ is reported by employing Eu^3+^ as a spectroscopic probe, for which X‐ray, neutron, and electron diffraction have confirmed the average long‐range structure with the space group *P*3_1_21. However, luminescence shows a lower local symmetry, as confirmed by the extended X‐ray absorption fine structure. By considering the reduced symmetry of the local structure, this work opens the door to a new class of understanding of the properties of materials.

Energy represents a better life for generations to come. For that lithium compounds have never attracted so much attention in the past until recently, driven by the requirements of energy‐storage and energy‐saving applications, such as Li‐ion batteries (LBs) and light‐emitting diodes (LEDs). Aiming at these applications, lithium‐containing silicates with the general formula ABC_2_X_4_, such as Li_2_SrSiO_4_,^1^, ^2^, ^3^, ^4^, ^5^, ^6^, ^7^, ^8^, ^9^, ^10^, ^11^ Li_2_EuSiO_4_,^12^ Li_2_BaSiO_4_,^13^ Li_2_CaSiO_4_,^14^ Li_2_MgSiO_4_,^15^ Li_2_ZnSiO_4_,^15^ Li_2_FeSiO_4_,^16^ Li_2_CoSiO_4_,^17^ and Li_2_MnSiO_4_,^17^, ^18^ have been extensively investigated. Among them, Li_2_SrSiO_4_ is a desirable host for LED phosphors, including the yellow emission of Eu^2+^,^5^, ^6^, ^7^ the blue emission of Ce^3+^,^8^ the white co‐emission of Ce^3+^ and Eu^2+^,^9^, ^10^, ^11^ and the multiband emission of Ce^3+^ and Pr^3+^ for plant growth,^3^ in Li_2_SrSiO_4_ and a promising optical coating for the cathodes of LBs.^1^

In 1998, Haferkorn first determined the crystal structure of Li_2_EuSiO_4_ and considered it isostructural with Li_2_SrSiO_4_.^12^ Later, several scholars carried out structure refinement of Li_2_SrSiO_4_ by taking Li_2_EuSiO_4_ as a starting model, confirming that Li_2_SrSiO_4_ crystallizes into the trigonal space group *P*3_1_21.^4^, ^5^, ^6^, ^7^ Although, in the structural model, the interatomic distances deviated significantly from those expected based on the ionic radii and bond valence sums,^19^ and the [SrO_8_] polyhedron presents an extremely distorted structure with a much larger eccentric distance and sphericity than expected.^11^ In 2010, Fukuda^19^ also revised this structure, based on synchrotron data, with space group *P*3_1_21. Nevertheless, the structural model of space group *P*3_1_21 (noted as the *P*3_1_21 model hereafter) possesses one Sr^2+^ site and thus cannot interpret the luminescence properties of Eu^2+^ and Ce^3+^ in Li_2_SrSiO_4_ in a straightforward way.^2^, ^3^, ^4^, ^5^, ^6^, ^7^, ^8^, ^9^, ^10^

Structures are always fundamental to understanding material properties.^20^, ^21^ However, the performance and mechanisms of materials are generally elucidated based on a static structure in regards to a standard structural model. However, in some situations, dynamic processes are not ignorable, such as the charging and recharging processes in batteries^22^ and the popular carburizing,^23^ boronizing,^24^ and natural aging^25^ treatments used to enhance the mechanical strength of steels, due to the transfer of small atoms, such as B, C, and N, and defects from one site to another. Li^+^ is the smallest and lightest alkali metal ion and can easily be incorporated into the interstitial sites in crystal lattices. The mobility and static displacement of small atoms may result in characteristic local symmetry breaking due to tiny distortions in the structure that do not propagate over long‐ranges.^26^

Symmetry is a beautiful and harmonic natural concept, but much of the changes in the world have been caused by symmetry breaking,^27^, ^28^ including the origin of species.^29^, ^30^ On one hand, the development of modern physics is filled with the history of symmetry that is constantly found, and on the other hand, symmetry breaking is continuously occurring,^28^ such as the violation of parity laws in weak interactions,^31^ CP (i.e., charge conjugation symmetry (C) and parity symmetry (P)) violation in the decay of neutral K‐mesons,^32^ spontaneous symmetry breaking in subatomic physics related to the Higgs mechanism,^33^ and spatial parity‐symmetry breaking of quantum phase transitions.^34^ Symmetry breaking does not imply that no symmetry is present; rather, the initial symmetry is lowered to a subgroup.^26^ Phase transitions and spontaneous symmetry breaking are widespread topics in condensed matter physics, and studies on spatial symmetry have greatly improved our understanding of materials.^35^, ^36^, ^37^ Yet, finding an observable quantity that is hidden under the superfine structure is still a big challenge in experiments.

According to Lee (the Nobel Laureate in Physics in 1957), “the root of all symmetry principles lies in the assumption that it is impossible to observe certain basic quantities.”^38^ In contrast, any discovery of symmetry breaking suggests the existence of a specific measurement.

According to symmetry breaking, forbidden transitions of electrons may occur due to a perturbation in the Hamiltonian. Therefore, fluorescence spectra originating from parity violation promisingly provides a probe for hyperfine interactions. In 1984, Zhang calculated the values of the Stark energy levels for ^7^F*_J_* splitting and the ^5^D_0_‐^7^F*_J_* transitions of the f^6^ configuration in 32 point groups, with consideration of the odd crystal field in Judd–Ofelt theory, and reported that the relationship between luminescence spectra and crystal structures could be used to investigate the local environments in crystal structures by doping trace amounts of Eu^3+^ (or Sm^2+^) into compounds as a probe.^39^ Later, this method was developed by improving the calculations for the electronic states and designing charts via the work of Binnemans,^40^ Görller‐Walrand,^41^ and Tanner^42^ to aid in the assignment of point symmetry. In addition to Eu^3+^, other rare‐earth ions, such as Dy^3+^ and Sm^3+^, have also been used as structural probes to study local site symmetry, but comparatively, Eu^3+^ is the best probe due to the large splitting of the ^7^F_1_ or ^7^F_2_ levels of Eu^3+^ in the visible wavelength region, which results in easily identified emission peaks.^43^, ^44^, ^45^ Moreover, the development of optical laser spectroscopy technologies have promisingly provided ultrafast‐response, high‐intensity, and high‐resolution tools for experimental studies.^46^

Society longs to find the beauty of a structure. Therefore, in this work, we report the discovery of symmetry breaking in small domains of Li_2_SrSiO_4_. Two Sr^2+^ sites in the Li_2_SrSiO_4_ structure with *P*3_1_21 symmetry are broken into *C*2 subgroups, as distinguished by employing Eu^3+^ as a spectroscopic probe. The difference between the two sites is ≈0.2 nm for the Eu^3+ 5^D_0_‐^7^F_0_ transition, which is out of the discernable range of X‐ray, electron and neutron diffraction, and nuclear magnetic resonance measurements but is sensitive to Eu^3+^ fluorescence spectra. This work demonstrates a facile, but powerful, optical tool to probe hyperfine structures and opens a door to identify new material properties and mechanisms by considering their reduced symmetry. Justifiably, symmetry breaking will not only exist in Li_2_SrSiO_4_ but also in other lithium compounds. Thereby, this discovery represents a landmark in exploring hyperfine structures with subgroups, which will improve our comprehension of modern physics and existing philosophy.

The XRD patterns of Ce^3+^, Eu^2+^, and Eu^3+^‐doped LiSrSiO_4_, shown in Figure S1 in the Supporting Information, match well with those of Li_2_EuSiO_4_ (JCPDS 47‐0120).^12^ Based on their ionic radii and charge balance, Ce^3+^ (103 pm) and Eu^2+^ (109 pm) should replace Sr^2+^ (112 pm) in the blue‐emitting Li_2_SrSiO_4_:Ce^3+^ and yellow‐emitting Li_2_SrSiO_4_:Eu^2+^ phosphors. Eu^3+^ should also occupy the Sr^2+^ site in Li_2_SrSiO_4_ despite its smaller radius (95 pm), as is known, e.g., from Eu^3+^‐doped Zn_2_SiO_4_ or SrSnO_3_ perovskites.^47^, ^48^, ^49^ Although a minority of the sites may be occupied,^50^ the luminescence of Eu^3+^ may be used as a spectroscopic probe for the symmetry of the initial coordination of Sr^2+^ in Li_2_SrSiO_4_.^40^

The asymmetrical shapes of the spectra in Figure S2a,b in the Supporting Information suggest that the emission peaks of Li_2_SrSiO_4_:Ce^3+^ and Li_2_SrSiO_4_:Eu^2+^ comprise more than one peak each, as can be observed in previous reports.^4^, ^5^, ^6^, ^7^, ^8^, ^9^, ^10^ These spectra can be fit with two Gaussian functions (Figure S2a,b, Supporting Information). For Ce^3+^, the doublet peaks can be attributed to transitions from the lowest 5d excited state to the ground substates of ^2^F_7/2_ and ^2^F_5/2_ (degenerate). However, this conclusion is not possible for Eu^2+^ because there is no spin‐orbit splitting for the ^8^S_7/2_ ground state of Eu^2+^. Nevertheless, emission peaks originating from different environments around Eu^2+^ cannot be excluded. However, there is only one Sr site in the *P*3_1_21 model of Li_2_SrSiO_4_.^12^, ^19^ To clarify the recognition of the spectral assignment, site occupation, and energy transfer of Ce^3+^ and Eu^2+^ in Li_2_SrSiO_4_, an accurate crystal structure should be confirmed first.^4^, ^5^, ^6^, ^7^, ^8^, ^9^, ^10^ An exact crystal structure is helpful for understanding the intrinsic properties of a material and further inspiring new applications.

**Figure** **1**a presents the full‐level ^5^D_0_‐^7^F*_J_* (*J* = 0, 1, 2, 3, and 4) emission spectra of Li_2_SrSiO_4_:Eu^3+^ with a nominal composition of Li_2_Sr_0.995_Eu_0.005_SiO_4_. The electric dipole transition of ^5^D_0_‐^7^F_2_ is parity‐forbidden, but it can potentially occur in a noninversion site by mixing with the opposite component. If allowed, its intensity would be far stronger than the intensity of the magnetic ^5^D_0_‐^7^F_1_ transition. Therefore, the strong ^5^D_0_‐^7^F_1_ emission in Figure 1a should come from Eu^3+^ that occupies an inversion or approximate inversion site. However, the Wyckoff positions 3*a* 12 (or 3*b* 19) of Sr in the *P*3_1_21 model have noninversion symmetry.

**Figure 1:**
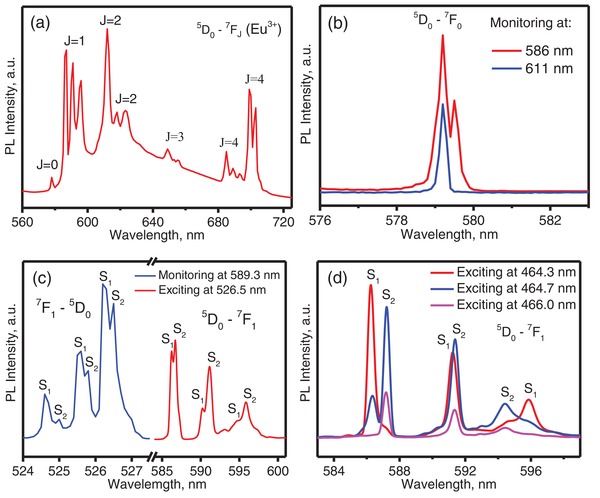
Low‐temperature emission and excitation spectra of Li_2_SrSiO_4_:Eu^3+^. a) Full‐spectrum emission under 393 nm excitation at 10 K, measured with a Fluorolog‐3‐Tau spectrometer; b) the ^5^D_0_‐^7^F_0_ transitions for the ^5^D_0_‐^7^F_1_ (586 nm) and ^5^D_0_‐^7^F_2_ (611 nm) emission; c) the ^5^D_0_‐^7^F_1_ emission excited at 526.5 nm and the ^7^F_0_‐^5^D_1_ excitation by monitoring at 589.3 nm; d) the ^5^D_0_‐^7^F_1_ emission by exciting the ^5^D_2_ level at 464.3, 464.7, and 466.0 nm, respectively. (b–d) were measured with an OPO at 20 K.

Figure 1b presents the ^5^D_0_‐^7^F_0_ transition as measured with an optical parametric oscillator (OPO) at 20 K. One peak at 579.2 nm can be observed by monitoring the ^5^D_0_‐^7^F_2_ emission at 611 nm, while two peaks at 579.2 and 579.4 nm can be observed by monitoring the ^5^D_0_‐^7^F_1_ emission at 586 nm. Theoretically, the number of peaks for the Eu^3+ 5^D_0_‐^7^F*_J_* transition will be no more than 2*J* + 1, where *J* is the total angular momentum. Thus, the number of peaks for the ^5^D_0_‐^7^F_0_ transition at each site, which is strictly forbidden by parity and spin rules due to Δ*J* = 0 for the transition from *J* = 0 to *J* = 0, should be no more than 1. Two peaks for the ^5^D_0_‐^7^F_0_ transition can be observed in Figure 1b, indicating the existence of two different environments of Eu^3+^ in Li_2_SrSiO_4_.

In addition to detection the number of sites, site symmetry could be probed by the ^5^D_0_‐^7^F_1_ transition, for which the splitting strongly depends on the symmetry. In the *P*3_1_21 model, the Sr^2+^ at the Wyckoff 3*a* 12 (or 3*b* 19) position has C_2_ symmetry. Thus, the ^5^D_0_‐^7^F_1_ emission should have 3 peaks if only one Sr^2+^ site with C_2_ symmetry exists in Li_2_SrSiO_4_.^39^, ^40^, ^41^, ^42^ However, six peaks, for both the ^5^D_0_‐^7^F_1_ emission and the ^7^F_1_‐^5^D_0_ excitation, are observed in Figure 1c. These peaks may be attributed to two different centers (S_1_ and S_2_), each with triplet emission. More than 3 peaks for the ^5^D_0_‐^7^F_1_ emission are observed upon exciting the ^5^D_2_ level at 464.3, 464.7, and 446.0 nm, as shown in Figure 1d, where the relative luminescence intensities of the two centers change with changing excitation wavelength, indicating multiple paths of energy relaxation from the ^5^D_2_ to ^5^D_1_ levels. From the above results, we can conclude that two different environments for Eu^3+^ exist in the crystal lattice of Li_2_SrSiO_4_.

The luminescence spectra suggest more than one local environment for Eu^3+^. To analyze this apparent disparity, the long‐range structure of Li_2_SrSiO_4_ was studied with X‐ray diffraction (XRD), synchrotron X‐ray diffraction (SXRD), and neutron diffraction (ND). By decreasing the symmetry of the *P*3_1_21 model, a hypothetical model with the translationengleiche subgroup *C*2 was derived and tentatively refined. No significant differences between the refined structures in the *C*121 and *P*3_1_21 space groups were found, either in the XRD or in the ND data (**Figure** **2**a–d).

**Figure 2:**
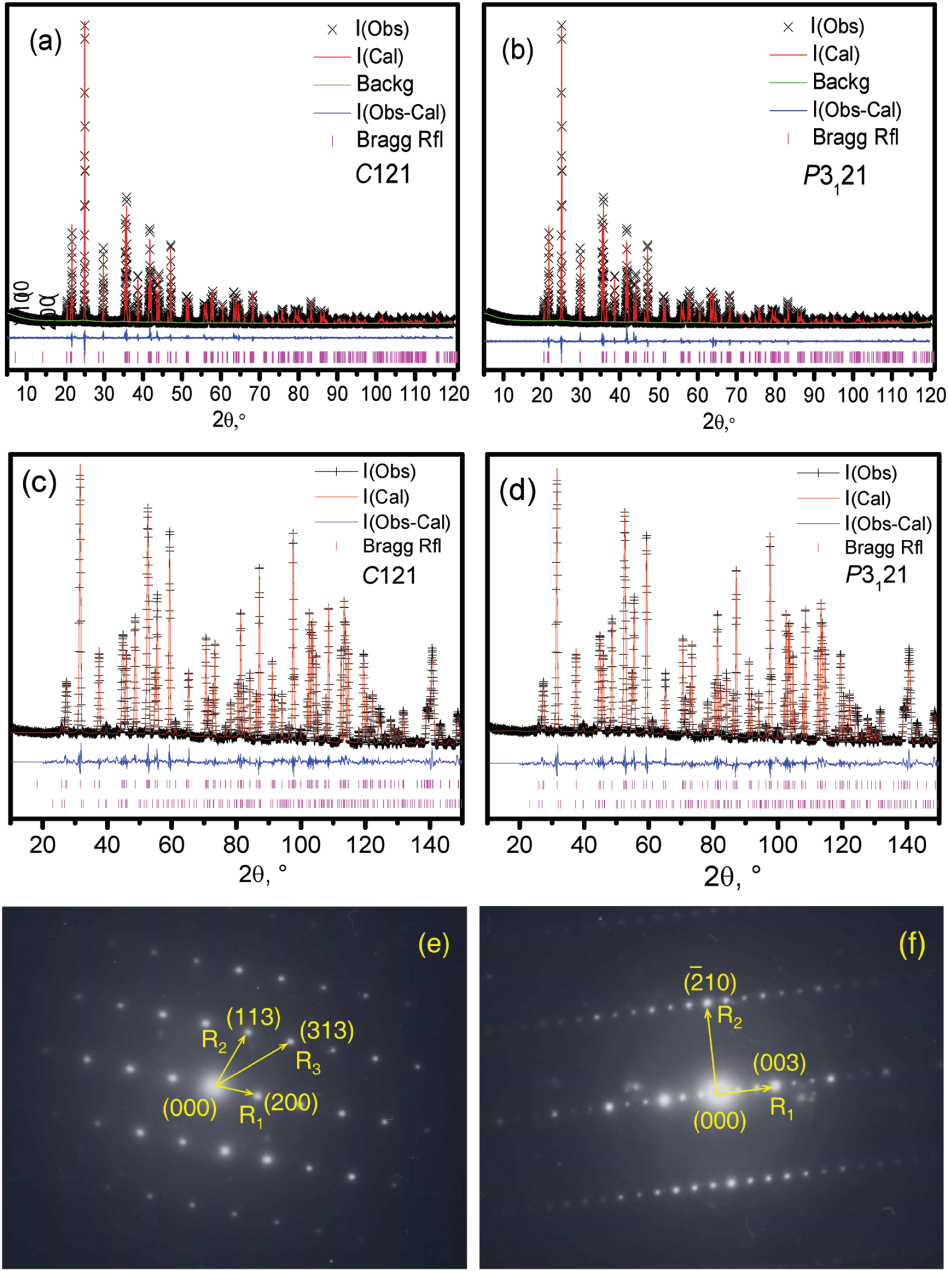
The Rietveld refinement of the crystal structure of Li_2_SrSiO_4_ with initial models in space groups a,c) *C*2 and b,d) *P*3_1_21 based on a,b) X‐ray diffraction and c,d) neutron diffraction; c,d) the second row of Bragg markers belongs to the secondary phase (1.4(1) wt%) of SrCO_3_ identified in the neutron diffraction data; e,f) the selected area electron diffraction patterns ($\left[03\bar{1}\right]$ and [350] zone axes) of Li_2_SrSiO_4_.

Systematically absent Bragg reflections of *P*3_1_21, such as the 001 and 002 diffractions, are allowed in *C*121 but were not observed in the XRD (Figure 2a,b), SXRD (Figure S3a,b, Supporting Information), and ND patterns (Figure 2c,d). Details regarding the comparison of the *C*2 and *P*3_1_21 models are included in Figures S4 and S5 in the Supporting Information. To check for possible phase transitions at low temperature, X‐ray diffraction data were collected at 12 K ≤ *T* ≤ 295 K and analyzed by Rietveld refinement. However, no new reflection peaks occurred, and no splitting or broadening of the reflection peaks was observed. Except for a slight lattice parameter decrease with decreasing temperature ( Table S1, Supporting Information), which can be attributed to regular thermal expansion effects, no significant changes in the crystal structure were detected. Therefore, we can conclude that no phase transition occurs down to 12 K and that the crystal structure is best described by the model of space group *P*3_1_21 for all investigated temperatures. Details for the methodology of the XRD, SXRD, and ND Rietveld refinement are given in Figures S3–S5 and Table S2 in the Supporting Information.

Figure 2e presents the selected area electron diffraction (SAED) pattern of the $\left[03\bar{1}\right]$ zone axis, which can be perfectly simulated with both the *C*2 and the *P*3_1_21 models (Figure S6, Supporting Information). For space group *P*3_1_21, kinematic theory only allows for 00*l* reflections with *l* = 3n, but these reflection may increase in intensity via dynamic electron diffraction. Nevertheless, the 003 peak intensity is much stronger than that of the other peaks in the [350] pattern (Figure 2f). In other words, Figures 2e,f shows that there is no superstructure with an enlarged unit cell, which could be another explanation for the multiple independent sites without lowering the point symmetry.

Solid‐state nuclear magnetic resonance (NMR) is sensitive to the local chemical environments of Li and Si atoms. In the *C*2 model, there are three sites of Li and two sites of Si, whereas there is only one site of Li and Si in the *P*3_1_21 model (Table S2, Supporting Information). However, only one type of chemical environment for Li and Si can be clearly discriminated in **Figure** **3**, in accordance with the *P*3_1_21 model. The chemical shifts of the ^7^Li and ^29^Si resonances are 0.5842 and −68 ppm, respectively, and the twin peaks at −94.2838 and 98.3730 ppm and −191.1380 and 194.2925 ppm are the first two and the second two spinning sidebands of ^7^Li. Since different sites can have the same chemical shift, the Li‐ and Si‐NMR results are inconclusive. Therefore, the NMR data are consistent with the higher symmetry, but they cannot exclude the lower symmetry.

**Figure 3:**
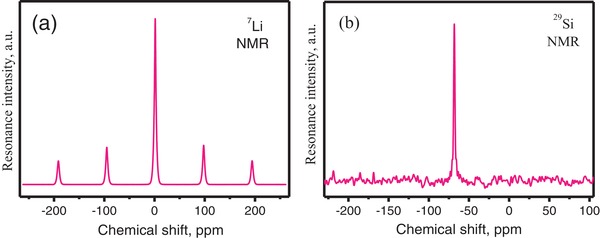
The NMR spectra of the a) Li and b) Si atoms in Li_2_SrSiO_4_.

Next, the local structure was examined with extended X‐ray absorption fine structure spectroscopy (EXAFS) to obtain information on the local coordination numbers, interatomic distances and possibly structural disorder. The Sr K‐edge EXAFS spectrum and its transformation in K‐space are shown in **Figures** **4**a,b, respectively. Starting from the *C*2 and *P*3_1_21 models that were hypothesized above and their 2 and 1 Sr‐atom sites, respectively, all 8 Sr–O bonds of the first coordination sphere of Sr cannot be fit at one time due to the large variations in the bond lengths. Instead, fitting was performed by classifying the 8 Sr–O bonds into two groups: a shorter group and a longer group. As shown in Figure 4c,d, the experimental curve with a main peak at 2.1 Å (no phase correction) can be more perfectly fit by the two sites of the *C*2 model than by the *P*3_1_21 model. In Figure 4c,d, the strongest peak at ≈2.1 Å in R‐space can be considered to correspond to the Sr–O bonds, and the second peak at 3.0 Å possibly corresponds to the main Sr–Si bond. The difference between the distances of the Fourier transform (FT) peaks in R‐Space and the real bond lengths is ≈0.5 Å for the first FT‐EXAFS shell of most metal–oxygen bonds. The real length of the Sr–O bonds can be obtained by fitting the experimental FT‐EXAFS curve. Table S3 in the Supporting Information gives the fitting results. As shown in Table S3 in the Supporting Information, the average lengths of the 8 Sr–O bonds in the *P*3_1_21 model are close to those in the *C*2 model, including both of the shorter Sr–O1 bonds (2.53 ± 0.02 Å for *C*2; 2.54 ± 0.02 Å for *P*3_1_21) and the longer Sr–O2 bonds (2.67 ± 0.02 Å for *C*2; 2.70 ± 0.02 Å for *P*3_1_21); the disorder factors of these bonds are within a reasonable range. When fit with the *C*2 model, the coordination number for the shorter and longer groups are both 4.0, and the total sum, 8.0, is consistent with the above results from XRD and SXRD; when fit with the *P*3_1_21 model, however, the coordination number for the shorter and longer groups are both 3.7, and the total sum, 7.4, is far below the coordination number, 8.0, resolved from the XRD and SXRD results. Therefore, the fit from *C*2 symmetry gives a more reasonable coordination number of *N* = 8.0, which is consistent with the crystal structure. Based on the coordination numbers (Table S3, Supporting Information), the analyses of the EXFAS spectra indicates a structure with two different environments of Sr in Li_2_SrSiO_4_ (as is theoretically possible for the *C*2 model).

**Figure 4:**
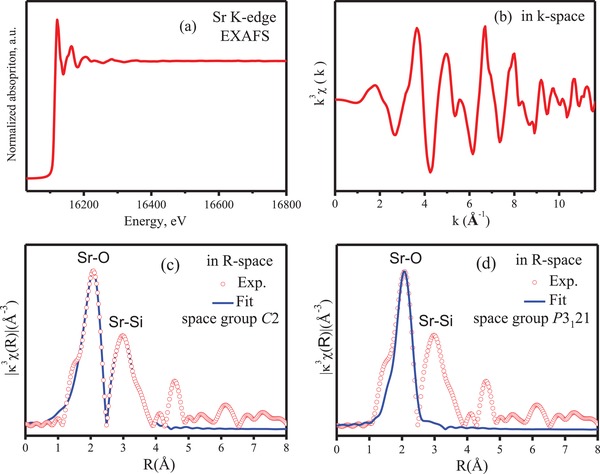
a) Sr K‐edge EXAFS and b) its Fourier transformation in k‐space; and the EXAFS spectra in R space fit with c) two sites and d) one site.

XRD, SXRD, ND, and SAED data indicate that the long‐range average structure of Li_2_SrSiO_4_ does not deviate significantly from that of the *P*3_1_21 model, whereas the cryogenic spectra of Eu^3+^ demonstrate that more than one Eu^3+^ site has to be taken into account. The diffraction data give an average structure model that projects the structure into a single unit cell, which requires a periodicity over an area larger than the coherence length of the radiation used. If local distortions are not long‐range ordered, they do not influence the space group assignment.

The multiple ^5^D_0_‐^7^F*_J_* transitions of Eu^3+^, either allowed or forbidden, are determined by the local symmetry. The cryogenic spectra of Eu^3+^ suggest that more than one Eu^3+^ site has to be considered. To explore the reason for the electronic structure, the charge deformation density of Li_2_SrSiO_4_ was obtained from first‐principles calculation as shown in **Figure** **5**, in which the blue color (negative value) indicates the loss of electrons and the red color (positive value) denotes gaining electrons. Although they are rather similar to each other, three distinct areas of electron density (labeled as hf‐1, hf‐2, and hf‐3 for the hyperfine structures) can be distinguished in Figure 5a for the *C*2 model, whereas these patterns merge (labeled as gb‐1, gb‐2, and gb‐3 for the gobbets) in Figure 5b for *P*3_1_21. It seems that the spectroscopic probe of Eu^3+^ also interacts with the shape of the electron density, a further aspect of the local structure in addition to its symmetry. The ab initio geometry optimization shows that the final enthalpies for *C*2 (−9251.08 eV) and *P*3_1_21 (−9250.79 eV) are very close to each other. Because the lower symmetry *C*2 model has a lower energy, local symmetry breaking seems to be possible due to the small energy barrier.

**Figure 5:**
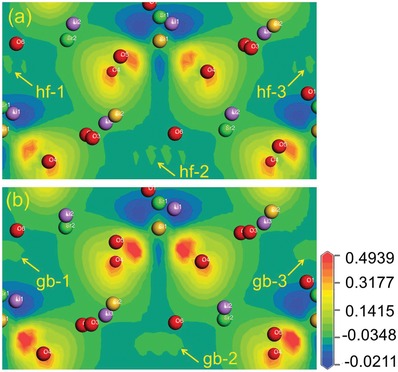
Charge density deformation of the a) *C*121 and b) *P*3_1_21 models for Li_2_SrSiO_4_.

Finally, we discuss the origin of symmetry breaking in Li_2_SrSiO_4_. Its crystal structure consists of [LiO_4_] and [SiO_4_] tetrahedrons. The periodical arrangement of three [SiO_4_] and six [LiO_4_] tetrahedrons forms a channel. Thus, the crystal structure of Li_2_SrSiO_4_ may be described by incorporating Sr atoms into the framework channels. There is one type of channel in *P*3_1_21 (**Figure** **6**a) but two in the hypothetical *C*2 model (Figure 6b). For instance, previous research has shown that with 20% of the Sr substituted with Ba, the structure of Li_2_(Sr_0.8_Ba_0.2_)SiO_4_ changes from trigonal to hexagonal,^51^ indicating that Li_2_SrSiO_4_ has a nonrigid structure. The tiny differences between the channels may lead to symmetry breaking from *P*3_1_21 into *C*2 (i.e., the symmetry breaking from one channel into two channels) since the superposition of local deviations results in *P*3_1_21 symmetry, described in Figure 6a,b. Figure 6c shows that by sharing Li–O bonds, the Sr atoms are located in different channels. Additionally, in either the *C*2 or the *P*3_1_21 model, all the Sr atoms are coordinated by 8 neighboring atoms, as shown in Figure 6d. For the comparison of the initial coordination of Sr with O and the Sr–O bond lengths in the *C*2 and *P*3_1_21 models, based on XRD data, the bond lengths of Sr1–O range from 2.553 to 2.691 Å, and Sr2–O range from 2.567 to 2.757 Å in the *C*2 model, wherein the variation amplitudes of the Sr1–O and Sr2–O bonds are 0.138 and 0.190 Å, respectively. However, the variation range of the Sr–O bond length in the *P*3_1_21 model is as small as 0.077 Å, changing from 2.576 to 2.653 Å. Because the effects of the surface, interface, crystal defects, etc., are always unavoidable, they cause a wide variation in the Sr–O bond lengths, which breaks the local symmetry of the Sr site in the *P*3_1_21 model, resulting in the appearance of two Sr sites in the *C*2 model.

**Figure 6:**
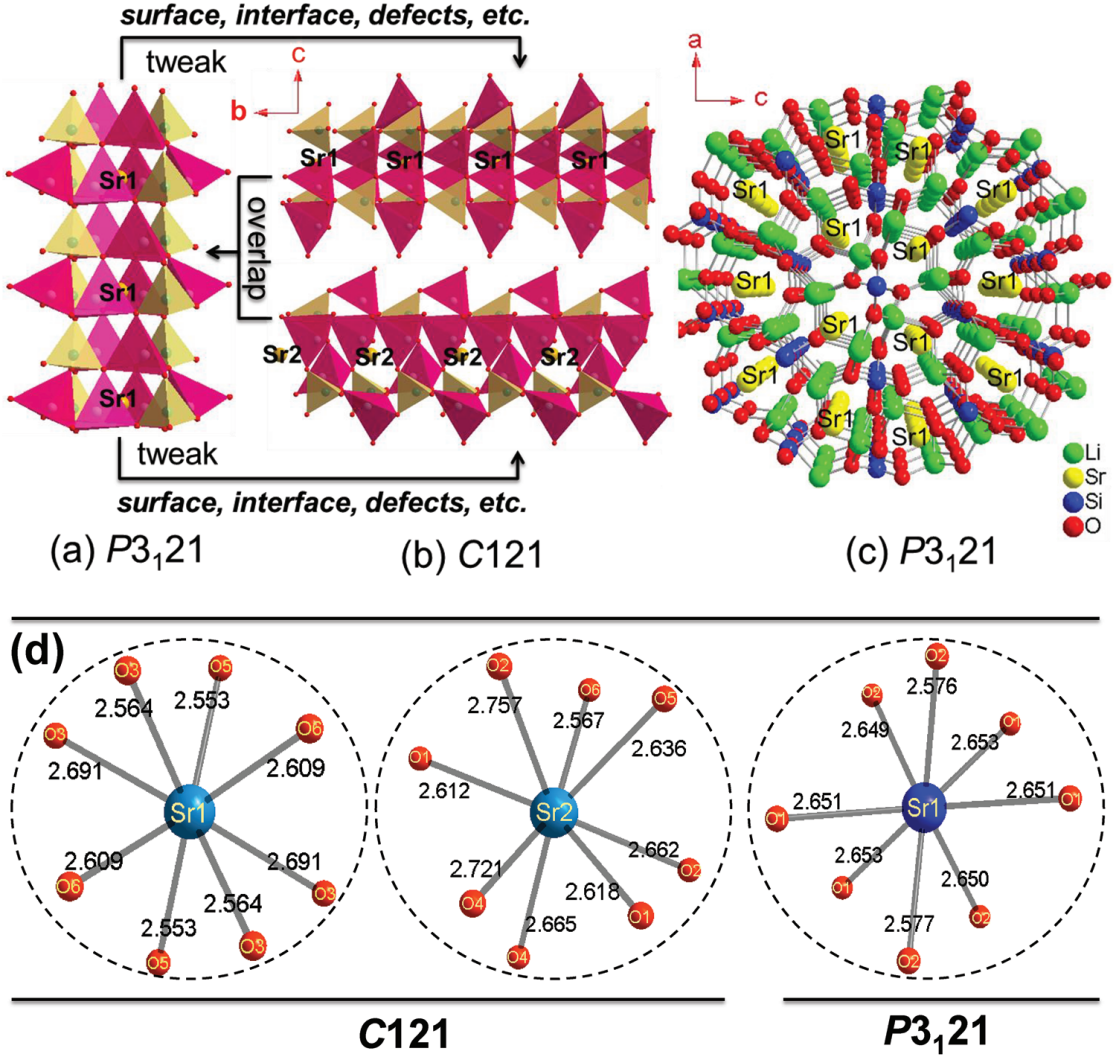
Schematic diagram of the symmetry breaking in the local structure of Li_2_SrSiO_4_. a) One channel comprised of [SiO_4_] and [LiO_4_] tetrahedrons in *P*3_1_21. b) Two independent channels in the hypothetical *C*2 model. c) The projection of the 3D structure of Li_2_SrSiO_4_ along the [010] axis. d) The coordination of Sr to neighboring O atoms and the Sr–O bond lengths (based on XRD data) in the *C*2 and *P*3_1_21 models.

By neglecting the minor differences in the local environments of the same types of atoms, including Li, Sr, Si, and O, the *C*2 structural model of Li_2_SrSiO_4_ can be approximated as the *P*3_1_21 model, which follows the same principle for ignoring higher order terms in the Taylor series expansion in mathematics. However, the results achieved in this work by employing Eu^3+^ as a spectroscopic probe suggest that the difference between Sr1 and Sr2 in Li_2_SrSiO_4_ is non‐negligible, as discussed above, regarding perturbations to the Hamiltonian of the system energy. This work not only distinguishes the hyperfine structures of Li_2_SrSiO_4_ but also presents a facile optical tool for detecting local structures and, more importantly, helps to explain crystal structures at the subgroup symmetry level. These discoveries will produce far‐reaching implications for understanding the properties and mechanisms of materials.

In summary, the phenomenon of a local structure with reduced symmetry, i.e., symmetry breaking, was discovered in Li_2_SrSiO_4_ by employing Eu^3+^ as a spectroscopic probe. The X‐ray, synchrotron X‐ray, and neutron diffraction results show that the long‐range average structure of Li_2_SrSiO_4_ is consistent with the symmetry of space group *P*3_1_21. However, the cryogenic spectra of Eu^3+^ suggest a lower local symmetry with the *C*2 space group. A comparison of the electron diffraction and NMR data suggest the presence of the *P*3_1_21 model but cannot exclude the possibility of the *C*2 model. Nevertheless, the Sr K‐edge EXAFS confirms that the local environment deviates from that of the *P*3_1_21 model but is similar to that of the *C*2 model. All of these evidences indicate that Li_2_SrSiO_4_ exists in a medium possessing characteristic symmetry in space group *P*3_1_21 for its long‐range structure and a lower symmetry local structure in space group *C*2. One site of Sr exists in *P*3_1_21, while two sites of Sr exist in the hypothetical *C*2 model. All the Sr atoms are eightfold coordinated irrespective of the symmetry model. Two sites of Sr could rationally explain the photoluminescent properties of Eu^2+^ and Eu^3+^ in Li_2_SrSiO_4_. The resolution of the Eu^3+ 5^D_0_‐^7^F_0_ emission for distinguishing the two sites of Sr^2+^ is 0.2 nm. Therefore, the powerful ability of a Eu^3+^ spectroscopic probe for detecting local hyperfine structures has been demonstrated; moreover, this work opens the door to elucidating the properties of lithium compounds by considering the reduced symmetry of the local structures. These results will produce far‐reaching implications for characterizing material structures and properties.

## Experimental Section

Samples were synthesized via a two‐step solid‐state reaction. Initially, Li_2_SrSiO_4_ was synthesized from SrCO_3_ (AR), Li_2_CO_3_ (AR), and SiO_2_ (99%). The residual SrCO_3_ in the final compound that had not been fully reacted was identified from ND in Figure 2c,d. Later, the samples were synthesized from SrO (AR), Li_2_O (AR), SiO_2_ (99%), Eu_2_O_3_ (99.99%), and CeO_2_ (99.99%) sources via the following process. First, stoichiometric amounts of the raw materials with a 3% excess of Li were thoroughly ground, and the mixture was heated at 500 °C for 2 h in air. Then, the mixture was ground again and sintered at 850 °C for 8 h. The blue‐emitting Li_2_SrSiO_4_:Ce^3+^ and yellow‐emitting Li_2_SrSiO_4_:Eu^2+^ phosphors were reacted under a 90% N_2_ + 10% H_2_ reductive atmosphere, and the red‐emitting Li_2_SrSiO_4_:Eu^3+^ phosphor was obtained in air. Conventional XRD was measured using a Rigaku D/max‐IIIA diffractometer with an X‐ray wavelength of 1.5418 Å. SXRD was recorded on the high‐energy X‐ray diffraction beamline SP12B1 of a Spring‐8 in Japan with an X‐ray wavelength of 0.6888 Å. The neutron diffraction was collected on a D20 diffractometer at the Institute Laue‐Langevin in Grenoble, France. The programs of GSAS^52^ and FullProf Suite^53^ were used to perform XRD, SXRD, and ND Rietveld refinement. Low‐temperature PL spectra at 10 K were collected with a Fluorolog‐3‐Tau (Jobin Yvon) spectrometer. High‐resolution (≈0.02 nm) PL spectra were recorded using an OPO equipped with a tunable laser system with a pulse duration of 7 ns at a 20 Hz repetition rate over an excitation wavelength range of 410–2200 nm. The electron diffraction pattern was recorded on a JEOL‐2010 transmission electron microscope operating at 200 kV with a camera length of 80 cm. The solid‐state MAS NMR spectra of ^7^Li and ^29^Si were collected on a Bruker Avance‐III, 400 MHz NMR instrument. The Sr K‐edge EXAFS was collected at the 01C1 beamline of the National Synchrotron Radiation Research Center (NSRRC) in Taiwan. The EXAFS data were fit using the FEFF8‐lite program. The ab initio geometry optimization and first‐principles calculations of the charge deformation density were performed using the CASTEP module of Materials Studio 7.0.

## Conflict of Interest

The authors declare no conflict of interest.

## Supporting information

Supplementary
