# Supplementary material for: Reduced Local Symmetry in Lithium Compound Li2SrSiO4 Distinguished by an Eu3+ Spectroscopy Probe
Source: Adv Sci (Weinh). 2019 May 20;6(16):1802126. doi: 10.1002/advs.201802126 (PMC6702644; doi:10.1002/advs.201802126)
Supplement: Supplementary file 1 — Supplementary [file ADVS-6-1802126-s001.pdf]

Copyright WILEY-VCH Verlag GmbH & Co. KGaA, 69469 Weinheim, Germany, 2019.

ADVANCED SCIENCE
Open Access

Supporting Information

for *Adv. Sci.*, DOI: 10.1002/advs.201802126

Reduced Local Symmetry in Lithium Compound Li_2SrSiO_4 Distinguished by an Eu^{3+} Spectroscopy Probe

Lei Chen, Peng Cheng, Zhao Zhang, Liangrui He, Yang Jiang, Guobao Li,* Xiping Jing, Yan'guang Qin, Min Yin, Ting-Shan Chan, Bin Hong, Shi Tao, Wangsheng Chu, Zhi Zhao, Haiyong Ni, Holger Kohlmann, and Oliver Oeckler**

Supporting Information

Reduced local symmetry in lithium compound Li_2SrSiO_4 distinguished by a Eu^{3+} spectroscopy probe

Lei Chen, Peng Cheng, Zhao Zhang, Liangrui He, Yang Jiang, Guobao Li*, Xiping Jing, Yan'guang Qin, Min Yin, Ting-Shan Chan, Bin Hong, Shi Tao, Wangsheng Chu, Zhi Zhao, Haiyong Ni, Holger Kohlmann, and Oliver Oeckler**

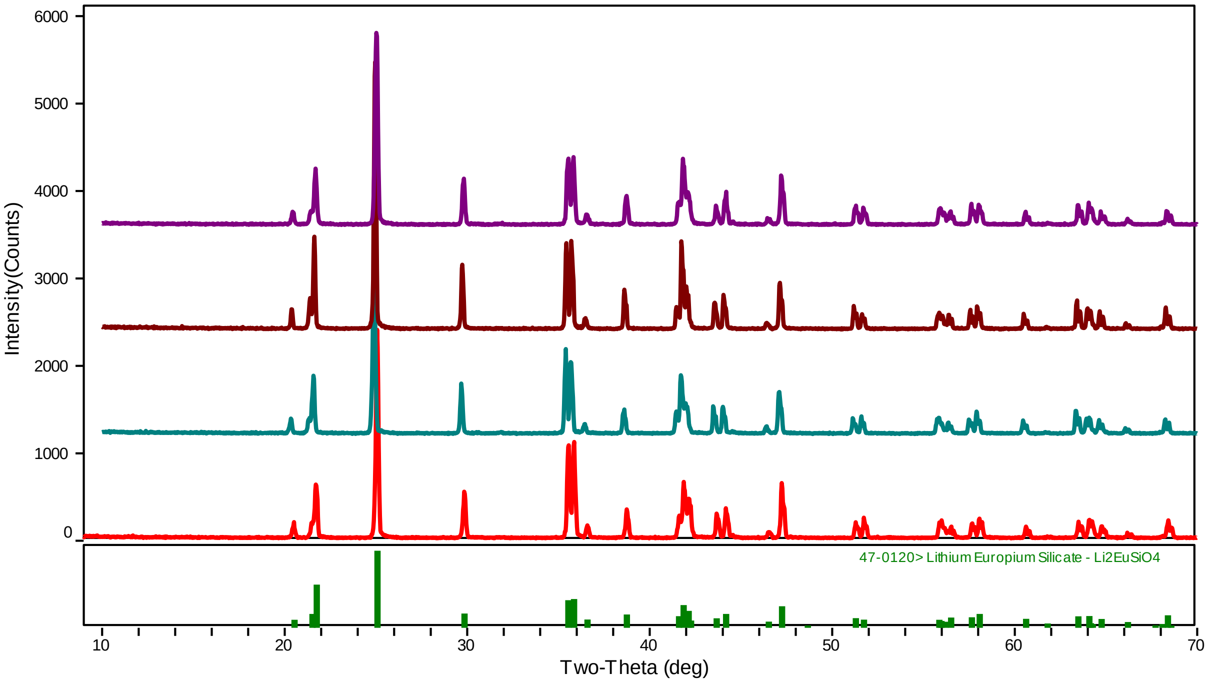

Supplementary Figure 1 XRD patterns of the Ce^{3+} (violet), Eu^{2+} (dark red), and Eu^{3+} (dark cyan)-doped Li_2SrSiO_4 phosphors and Li_2SrSiO_4 host (red) compared with JCPDS 47-0120 (Li_2EuSiO_4). The dopant concentrations of Ce^{3+}, Eu^{2+}, and Eu^{3+} are 0.008, 0.005 and 0.005 mol ratio of Sr^{2+}, respectively.

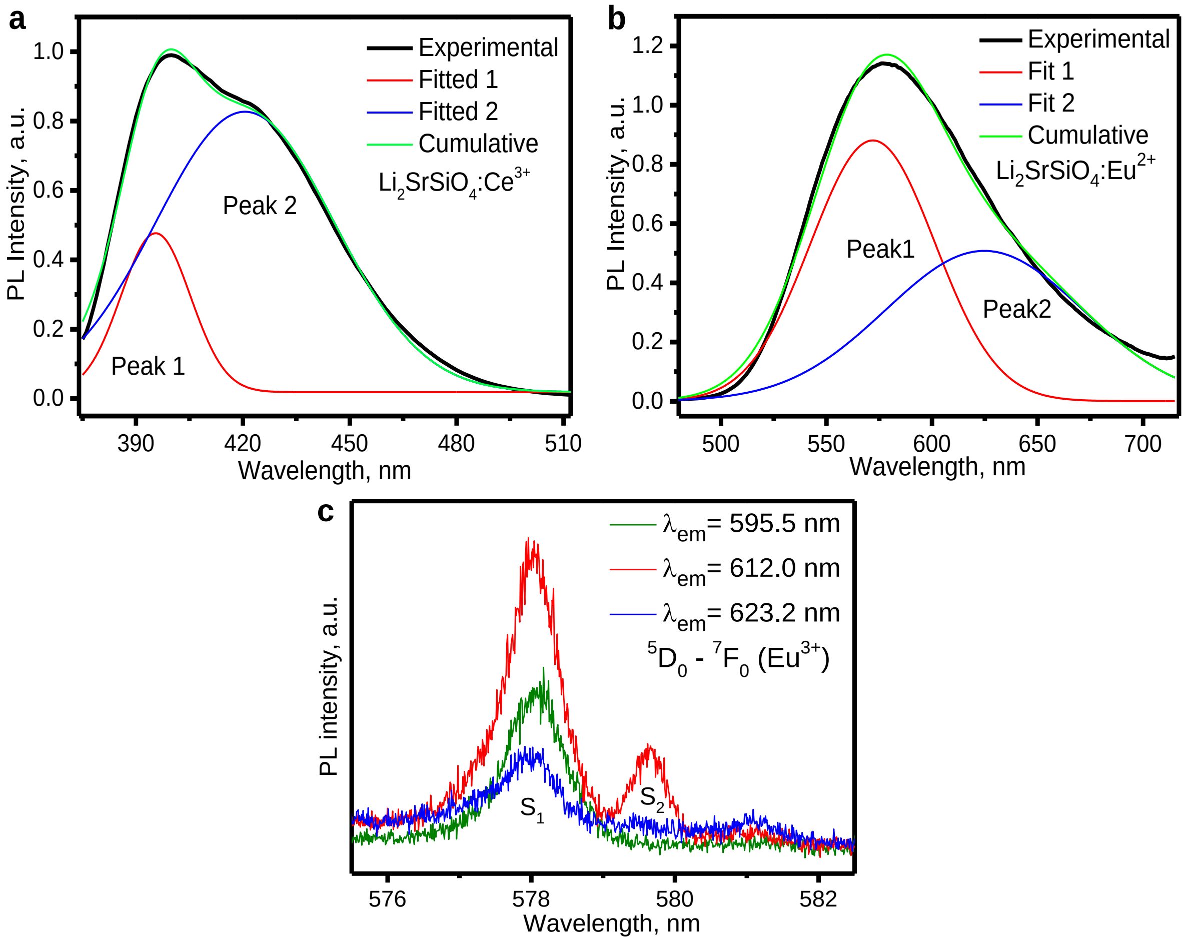

Supplementary Figure 2 Emission spectra of the Ce^{3+}, Eu^{2+}, and Eu^{3+} -doped Li_2SrSiO_4 phosphors. a) emission spectra of $Li_2Sr_{0.992}Ce_{0.008}SiO_4$ (Li_2SrSiO_4:Ce^{3+}), fit with two Gaussian functions; b) emission spectra of $Li_2Sr_{0.995}Eu_{0.005}SiO_4$ (Li_2SrSiO_4:Eu^{2+}), fit with two Gaussian functions; c) the $^5D_0$-$^7F_0$ excitation spectra of Li_2SrSiO_4:Eu^{3+} measured with a fluorescence spectrometer by monitoring the $^5D_1$ level at 595.5 nm and the $^5D_2$ level at 612.0 and 623.2 nm at 10 K.

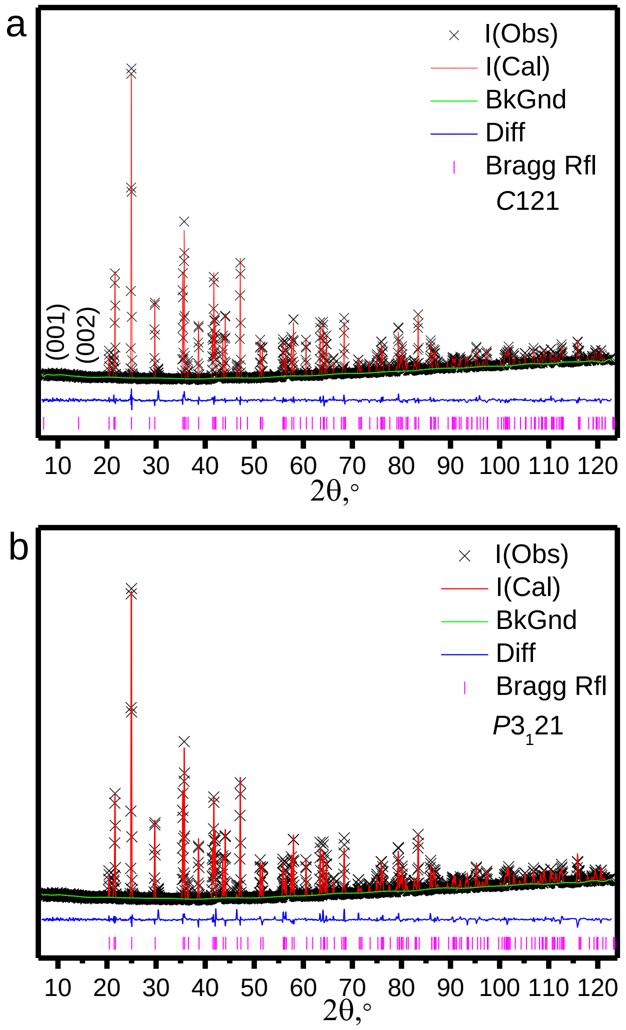

Supplementary Figure 3 Rietveld refinement of the crystal structure of Li_2SrSiO_4 based on synchrotron X-ray diffraction (SXRD) data by adopting space groups *C*121 and *P*$3_1$21 as the initial models in a) and b), respectively.

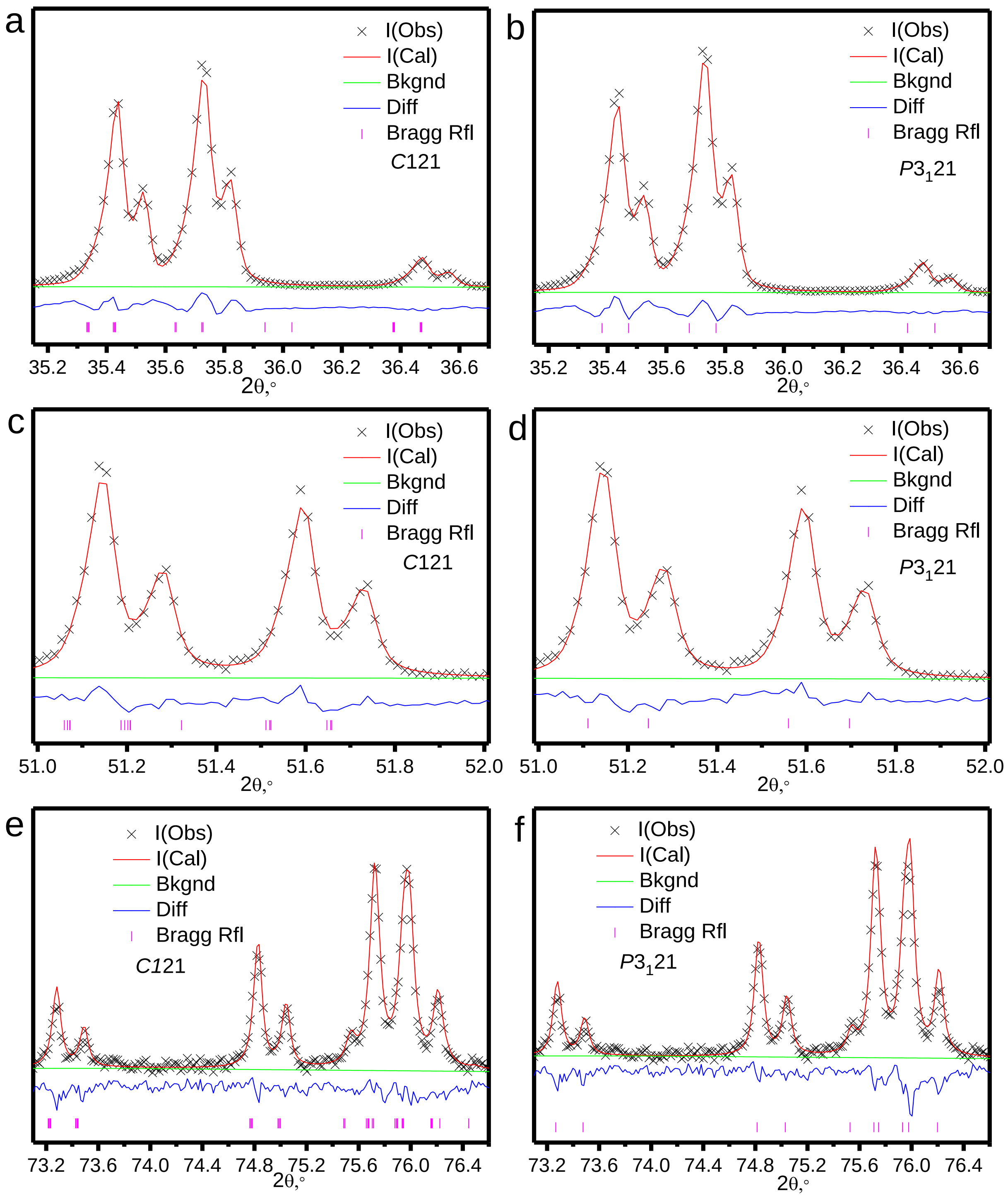

Supplementary Figure 4 Comparison of the *C*121 model a, c, e) with the $P3_121$ b, d, f) model for the Rietveld refinement of the crystal structure of Li_2SrSiO_4 based on X-ray diffraction (XRD) data with amplified patterns in the 2θ range of 35.15-36.7° a, b), 51-52° c, d), and 73.1-76.6° e. f).

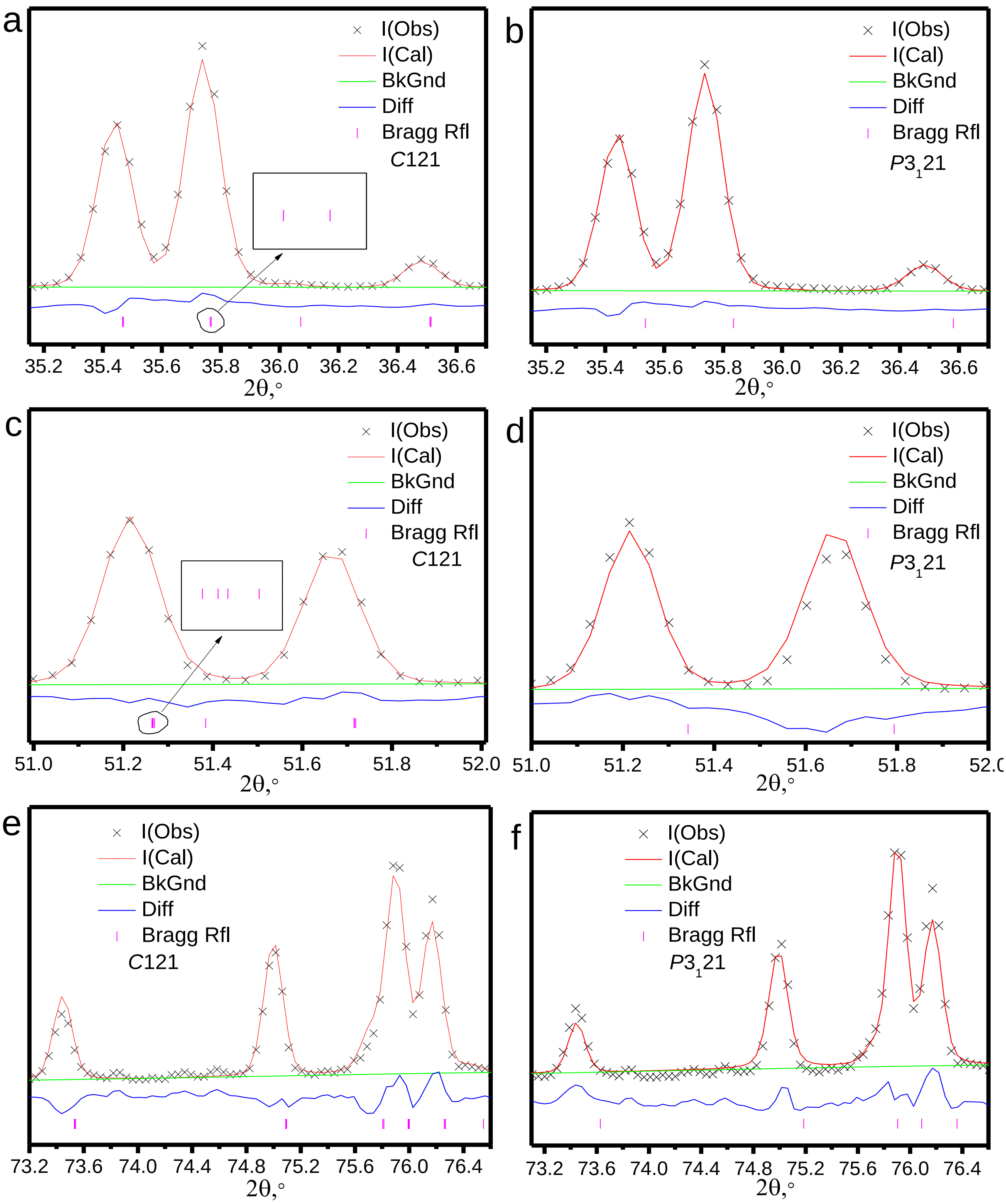

Supplementary Figure 5 Comparison of the *C*121 model a, c, e) with the *P*$3_1$21 b, d, f) model for the Rietveld refinement of the crystal structure of Li_2SrSiO_4 based on synchrotron X-ray diffraction (SXRD) data with amplified patterns in the 2θ range of 35.15-36.7°a, b), 51-52° c, d), and 73.1-76.6° e. f).

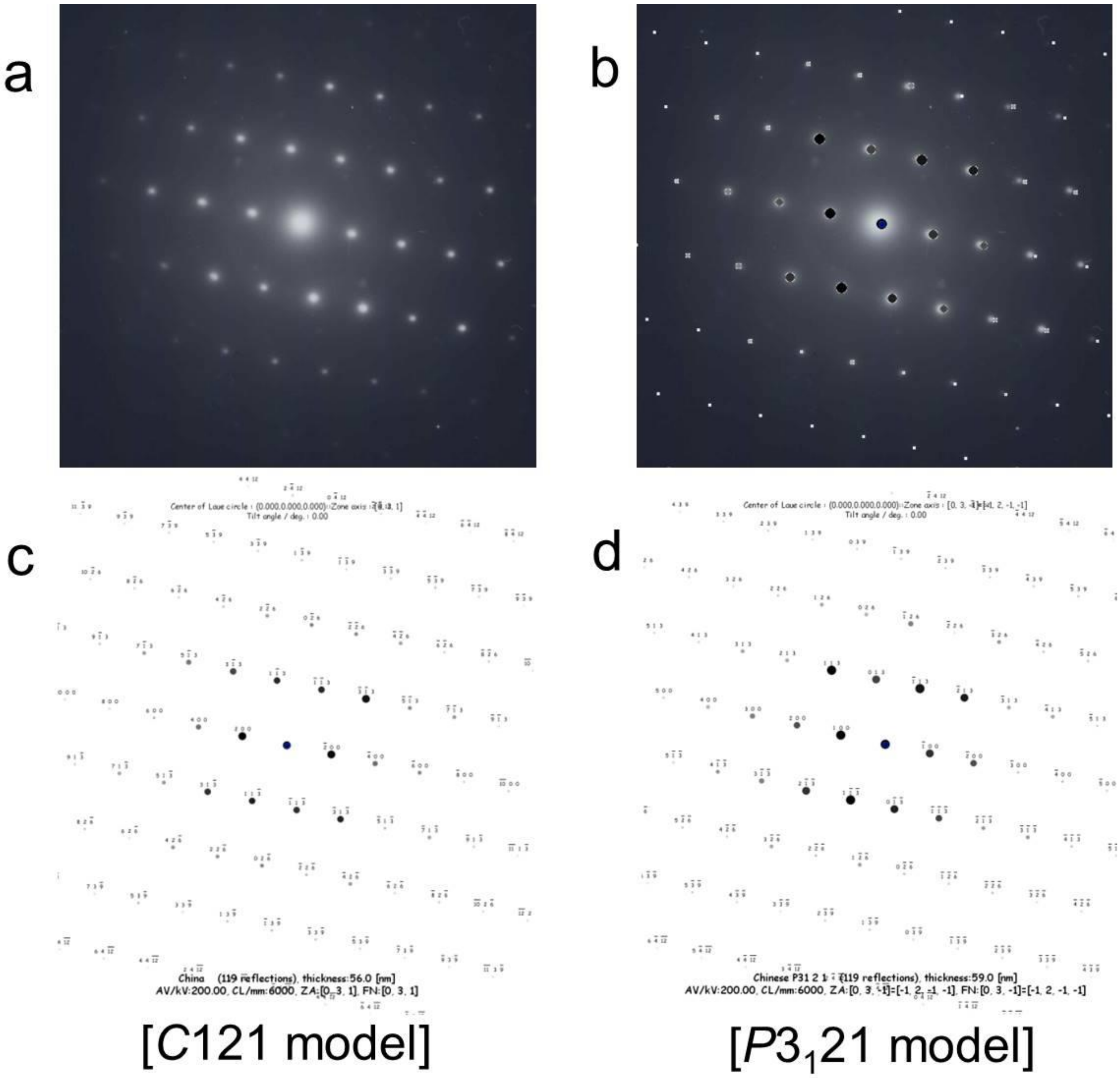

Supplementary Figure 6 Simulation of the selected area electron diffraction (SAED) patterns of Li_2SrSiO_4 for the structural models of *C*121 a, c) and $P3_121$ b, d) (top right image is overlain with a section of the calculated pattern).

Supplementary Table 1 The lattice parameters of Li_2SrSiO_4 for 12 K ≤ T ≤ 295 K

**Temperature (K)**	***a* (pm)**	***c* (pm)**
295	501.683(9)	1244.09(2)
200	501.245(8)	1243.07(2)
100	500.857(8)	1242.23(2)
50	500.771(8)	1242.07(2)
12	500.754(8)	1242.03(2)

Supplementary Table 2 The atomic parameters and phase data of Li_2SrSiO_4 derived from the Rietveld refinement of the crystal structure of Li_2SrSiO_4 based on the XRD (a, b), SXRD (c, d), and ND (e, f) data by employing space groups *C*121 (a, c, e) and $P3_121$ (b, d, f) as the initial models.

(a) Rietveld refinement of the crystal structure of Li_2SrSiO_4 based on the XRD data by employing space group *C*121 as the initial model

**Atom**	**Ox.**	**Wyck.**	**Symmetry**	**x/a**	**y/b**	**z/c**	**U [Å^2]**
Li1	1	4*c*	1	0.041(8)	0.356(12)	0.748(5)	0.01
Li2	1	4*c*	1	0.324(9)	0.427(11)	0.055(4)	0.049
Li3	1	4*c*	1	0.196(8)	0.734(9)	0.433(5)	0.01
Sr1	2	2*a*	2	0	0.4110(24)	0	0.0358
Sr2	2	4*c*	1	0.2064(5)	0.7761(22)	0.6654(4)	0.009
Si1	4	2*b*	2	0	0.2389(25)	1/2	0.001
Si2	4	4*c*	1	0.1473(11)	0.845(4)	0.1711(10)	0.0238
O1	-2	4*c*	1	0.0218(31)	0.462(5)	0.3832(17)	0.01
O2	-2	4*c*	1	0.2645(30)	0.266(5)	0.735(1)	0.01
O3	-2	4*c*	1	0.2252(30)	0.709(6)	0.0549(16)	0.01
O4	-2	4*c*	1	0.1256(25)	0.086(4)	0.5017(15)	0.01
O5	-2	4*c*	1	0.3607(21)	0.690(5)	0.8437(19)	0.01
O6	-2	4*c*	1	0.4441(22)	0.235(6)	0.1585(15)	0.01

Formula sum: Li_2O_4SiSr

Formula weight: 193.58 g/mol

Crystal system: monoclinic

Space group: *C*121 (5)

Cell parameters: a=8.7170(2) Å, b=5.0321(1) Å, c=12.4780(1) Å, β=89.99(0)°

Cell ratios: a/b = 1.7323; b/c = 0.4033; c/a=1.4314

Cell volume: 547.34(1) Å^3

Formula units per cell, Z = 6

Calc. density: 3.5235(2) g/cm^3

Person code: mC48; Formula type: NOP2Q4

χ^2 =3.034

wRp =6.72%

Rp = 5.18%

(b) The Rietveld refinement of the crystal structure of Li_2SrSiO_4 based on the XRD data by employing space group $P3_121$ as the initial model.

**Atom**	**Ox.**	**Wyck.**	**Symmetry**	**x/a**	**y/b**	**z/c**	**U [Å^2]**
Li1	1	6*c*	1	0.4105(32)	0.0754(24)	0.0795(9)	0.0160(31)
Sr1	2	3*b*	2	0	0.41799(25)	1/6	0.0167(3)
Si1	4	3*a*	2	0.7229(6)	0.7229(6)	0	0.0191(9)
O1	-2	6*c*	1	0.5457(13)	0.5097(9)	0.10774(25)	0.0155(10)
O2	-2	6*c*	1	0.7635(10)	0.0659(10)	0.0026(7)	0.0155(10)

Formula sum: Li_2O_4SiSr
Formula weight: 193.58 g/mol
Crystal system: trigonal
Space group: $P3_121$ (152)
Cell parameters: a = 5.033(0) Å; c = 12.4795(1) Å
Cell ratios: a/b=1.0000, b/c=0.4033, c/a=2.4795
Cell volume: 273.76(0) Å^3
Formula units per cell, Z = 3
Calc. density: 3.52233, g/cm^3
Person code: hp27
Formula type: NO2p2Q4

$\chi 2$ =3.120
wRp =6.83%
Rp = 5.17%

(c) Rietveld refinement of the crystal structure of Li_2SrSiO_4 based on the SXRD data by employing space group *C*121 as the initial model.

**Atom**	**Ox.**	**Wyck.**	**Symmetry**	**x/a**	**y/b**	**z/c**	**U [Å^2]**
Li1	1	4*c*	1	0.0847(28)	0.510(6)	0.7016(19)	0.033
Li2	1	4*c*	1	0.2070(28)	0.402(4)	0.0844(15)	0.0194
Li3	1	4*c*	1	0.090(4)	0.737(6)	0.3607(20)	0.0463
Sr1	2	2*a*	2	0	0.4694(19)	0	0.0005
Sr2	2	4*c*	1	0.2086(8)	0.8472(19)	0.6641(6)	-0.0001
Si1	4	2*b*	2	0	0.3124(34)	1/2	0.0558
Si2	4	4*c*	1	0.1436(11)	0.8993(30)	0.1675(12)	0.0138
O1	-2	4*c*	1	0.0185(19)	0.5891(27)	0.3805(8)	0.0828
O2	-2	4*c*	1	0.292(5)	0.317(7)	0.7470(16)	0.3018
O3	-2	4*c*	1	0.2241(14)	0.8405(31)	0.0531(8)	0.0635
O4	-2	4*c*	1	0.1303(16)	0.0784(22)	0.4849(9)	0.0524
O5	-2	4*c*	1	0.3350(24)	0.6800(33)	0.8101(14)	0.1467
O6	-2	4*c*	1	0.4618(17)	0.2148(23)	0.1419(11)	0.0763

Formula sum: Li_2O_4SiSr

Formula weight: 193.58 g/mol

Crystal system: monoclinic

Space group: *C*121 (5)

Cell parameters: a=8.6836(2) Å, b=5.0138(1) Å, c=12.4317(2) Å, β=89.99(0)°

Cell ratios: a/b = 1.7319; b/c = 0.4033; c/a=1.4316

Cell volume: 541.25(2) Å^3

Formula units per cell, Z = 6

Calc. density: 3.56319 g/cm^3

Person code: mC48

Formula type: NOP2Q4

χ^2 =3.034

wRp =6.72%

Rp = 5.18%

(d) Rietveld refinement of the crystal structure of Li_2SrSiO_4 based on the SXRD data by employing space group $P3_121$ as the initial model.

**Atom**	**Ox.**	**Wyck.**	**Symmetry**	**x/a**	**y/b**	**z/c**	**U [Å^2]**
Li1	1	6*c*	1	0.573(9)	-0.208(21)	0.157(6)	0.7043
Sr1	2	3*b*	2	0	0.4180(4)	1/6	0.0048
Si1	4	3*a*	2	0.7248(9)	0.7248(9)	0	0.0346
O1	-2	6*c*	1	0.5365(14)	0.5303(15)	0.1061(3)	0.1926
O2	-2	6*c*	1	0.7372(9)	0.0631(10)	-0.0168(4)	0.1728

Formula sum: Li_2O_4SiSr
Formula weight: 193.58 g/mol
Crystal system: trigonal
Space group: $P3_121$ (152)
Cell parameters: a = 5.0121(1) Å; c = 12.4279(1) Å — $\chi2$ =3.120
Cell ratios: a/b=1.0000, b/c=0.4033, c/a=2.4796 — wRp =6.83%
Cell volume: 270.37(1) Å^3 — Rp = 5.17%
Formula units per cell, Z = 3
Calc. density: 3.5665 g/cm^3
Person code: hp27
Formula type: NO2p2Q4

(e) Rietveld refinement of the crystal structure of Li_2SrSiO_4 based on the neutron diffraction (ND) data by employing space group *C*121 as the initial model.

**Atom**	**Ox.**	**Wyck.**	**Symmetry**	**x/a**	**y/b**	**z/c**	**U [Å^2]**
Li1	1	4*c*	1	0.0365(35)	0.0760(71)	0.7572(18)	0.0068(22)
Li2	1	4*c*	1	0.2774(34)	0.0664(60)	0.0881(19)	U_{Li1}
Li3	1	4*c*	1	0.3322(38)	0.1572(86)	0.5882(22)	U_{Li1}
Sr1	2	2*a*	2	0	0.0000(-)	0	0.0096(6)
Sr2	2	4*c*	1	0.2904(16)	0.1262(44)	0.3301(10)	U_{Sr1}
Si1	4	2*b*	2	0	0.1324(72)	1/2	0.0073(11)
Si2	4	4*c*	1	0.1413(16)	0.5419(54)	0.1649(12)	U_{Si1}
O1	-2	4*c*	1	0.1181(15)	0.2416(47)	0.1686(71)	0.0095(4)
O2	-2	4*c*	1	0.1560(21)	0.3358(41)	0.5085(7)	U_{O1}
O3	-2	4*c*	1	0.2417(15)	0.6227(37)	0.2773(9)	U_{O1}
O4	-2	4*c*	1	0.2255(16)	0.6796(42)	0.0662(9)	U_{O1}
O5	-2	4*c*	1	0.4340(18)	0.1940(50)	0.1722(10)	U_{O1}
O6	-2	4*c*	1	0.5159(17)	0.4518(44)	0.3933(12)	U_{O1}

Formula sum: Li_2O_4SiSr
Formula weight: 193.58 g/mol
Crystal system: monoclinic
Space group: *C*121 (5)
Cell parameters: a=8.6965(5) Å, b=5.0229(2) Å, c=12.4525(2) Å, β=90.023(5)°
Cell ratios: a/b = 1.7314; b/c = 0.4034; c/a=1.4319
Cell volume: 543.94(4) Å^3
Formula units per cell, Z = 6
Calc. density: 3.546 g/cm^3
Person code: mC48
Formula type: NOP2Q4

χ^2 =6.67
wRp =3.54%
Rp = 2.74%

(f) Rietveld refinement of the crystal structure of Li_2SrSiO_4 based on the ND data by employing space group $P3_121$ as the initial model.

**Atom**	**Ox.**	**Wyck.**	**Symmetry**	**x/a**	**y/b**	**z/c**	**U [Å^2]**
Li1	1	6*c*	1	0.0739(11)	0.4117(12)	0.4195(5)	0.0133(11)
Sr1	2	3*a*	2	0.4176(3)	0	1/3	0.0096(5)
Si1	4	3*b*	2	0.2775(6)	0	5/6	0.0079(8)
O1	-2	6*c*	1	0.0355(3)	0.4841(3)	0.0606(1)	0.0095(4)
O2	-2	6*c*	1	0.3051(3)	0.2372(3)	0.1707(2)	0.0094(4)

Formula sum: Li_2O_4SiSr

Formula weight: 193.58 g/mol

Crystal system: trigonal

Space group: $P3_121$ (152)

Cell parameters: a = 5.02207(7) Å; c = 12.4530(2) Å — $\chi2$ =7.22

Cell ratios: a/b=1.0000, b/c=0.4033, c/a=2.4797 — wRp =3.70%

Cell volume: 270.001(7) Å^3 — Rp = 2.87%

Formula units per cell, Z = 3

Calc. density: 3.5714, g/cm^3

Person code: hp27

Formula type: NO2p2Q4

Supplementary Table 3 The average bond lengths and the coordination numbers of the Sr atoms in the Li_2SrSiO_4 structure fitted from the Sr K-edge EXAFS.

Sample	Pair	N	R (Å)	σ^2 (×10^{-3} Å^2)
Space group *C*121	Sr-O1	4.0 ± 0.5	2.53 ± 0.02	5.8 ± 0.6
	Sr-O2	4.0 ± 0.5	2.67 ± 0.02	4.1 ± 0.5
Space group *P*$3_1$21	Sr-O1	3.7 ± 0.5	2.54 ± 0.02	4.0 ± 0.5
	Sr-O2	3.7 ± 0.5	2.70 ± 0.02	5.1 ± 0.6
